# Toxicity of Three Optical Brighteners: Potential Pharmacological Targets and Effects on *Caenorhabditis elegans*

**DOI:** 10.3390/toxics12010051

**Published:** 2024-01-09

**Authors:** Isel Castro-Sierra, Margareth Duran-Izquierdo, Lucellys Sierra-Marquez, Maicol Ahumedo-Monterrosa, Jesus Olivero-Verbel

**Affiliations:** 1 Environmental and Computational Chemistry Group, School of Pharmaceutical Sciences, Zaragocilla Campus, University of Cartagena, Cartagena 130014, Colombia; icastros@unicartagena.edu.co (I.C.-S.); mdurani@unicartagena.edu.co (M.D.-I.); lsierram@unicartagena.edu.co (L.S.-M.); 2 Natural Products Group, School of Pharmaceutical Sciences, Zaragocilla Campus, University of Cartagena, Cartagena 130014, Colombia; mahumedom@unicartagena.edu.co

**Keywords:** optical brighteners, toxicity, docking, *Caenorhabditis elegans*

## Abstract

Optical brighteners (OBs) have become an integral part of our daily lives and culture, with a growing number of applications in various fields. Most industrially produced OBs are derived from stilbene, which has been found in environmental matrices. The main objectives for this work are as follows: first, to identify protein targets for DAST, FB-28, and FB-71, and second, to assess their effects in some behaviors physiologic of *Caenorhabditis elegans*. To achieve the first objective, each OB was tested against a total of 844 human proteins through molecular docking using AutoDock Vina, and affinities were employed as the main criteria to identify potential target proteins for the OB. Molecular dynamics simulations took and validated the best 25 docking results from two protein databases. The highest affinity was obtained for the Hsp70-1/DAST, CD40 ligand/FB-71, and CD40 ligand/FB-28 complexes. The possible toxic effects that OBs could cause were evaluated using the nematode *C. elegans*. The lethality, body length, locomotion, and reproduction were investigated in larval stage L1 or L4 of the wild-type strain N2. In addition, transgenic green fluorescent protein (GFP) strains were employed to estimate changes in relative gene expression. The effects on the inhibition of growth, locomotion, and reproduction of *C. elegans* nematodes exposed to DAST, FB-71, and FB-28 OBs were more noticeable with respect to lethality. Moreover, an interesting aspect in OB was increased the expression of *gpx-4* and *sod-4* genes associated with oxidative stress indicating a toxic response related to the generation of reactive oxygen species (ROS). In all cases, a clear concentration-response relationship was observed. It is of special attention that the use of OBs is increasing, and their different sources, such as detergents, textiles, plastics, and paper products, must also be investigated to characterize the primary emissions of OBs to the environment and to develop an adequate regulatory framework.

## 1. Introduction

Worldwide, the detergent industry is one of the most consolidated industries and is characterized by its dynamism and the permanent appearance of new products, many resulting from innovation in different fields of application [1]. This has caused continuous growth in this industry, which goes hand in hand with increasing population and quality of life for different socioeconomic sectors [2,3]. This has, in turn, caused an increase in the demand for detergents, especially in personal care and home care products, sectors that comprise approximately 56% of the world’s production of detergents [4]. Moreover, there is a high demand for cleanliness and whiteness appearance by humans, forcing the use of additives that may help achieve this goal [5].

Detergents are one of the most notable fingerprints of the Anthropocene, in particular due to their high rate of consumption [6,7]. According to Fortune Business Insights, every year, the world consumes 78.5 M tons of these chemicals, some of which may reach rivers, swamps and oceans [8,9]. In general, detergents are made up of a complex mixture of chemicals, including surfactants, bleaching agents, enzymes, and other additives [10], including dispersing agents and optical brighteners (OBs) [11]. This last type of chemical is quite unknown by the general public despite permanent exposure to direct contact with these materials. OBs are frequently detected in wastewater, both domestic and industrial, with few reports on toxicological effects in biota, aggravated by their lack of biodegradability and capacity to bioaccumulate, becoming a global environmental problem [12,13].

From a chemical perspective, OBs are organic compounds that normally contain at least one highly substituted aromatic ring, with structures with many double bonds that, when activated by ultraviolet light, manifest fluorescence, leading to the emission of an additional amount of light within the visible wavelengths [14]. This occurs because known OBs contain aromatic components or pseudoaromatic (unsaturated) heterocyclic groups linked together by direct bonds or by small bridges, such as -CO-, -NHCO-, -CH=N-, or -CH=CH-. From the physicochemical point of view, OBs have an extensive resonance or π electron system, which can be excited in the 340–400 nm range—that is, they can absorb UV light and emit visible (blue–violet) light; thus, they can be classified as fluorescent systems, particularly in systems derived from stilbenes, coumarins, and pyrazolines [15]. As a result, these compounds have the ability to generate a visual effect of whitening and color enhancement, the main reason for which they are widely used in the detergent, textile, paper, and plastic industries. The most commonly used OBs are derivatives of anionic diamino stilbene (DAS), such as DNS and DAST [16], or derivatives of distyryl biphenyl (DSBP), such as Tinopal CBS-X [17].

Among the compounds listed above, DAST, FB-71, and FB-28 are commonly used in commercial detergents, including baby clothes, and are on the EPA’s control list. The environmental toxicity of detergents, as well as their components, has been evaluated employing a diversity of biological models, such as *Laeonereis culveri* [18], *Ctenopharyngodon idella* [19], and *Caenorhabditis elegans* [20], among others. In this study, *Caenorhabditis elegans* was used as a model because it offers several advantages in terms of versatility, reproducibility, time to obtaining results, and low cost [21,22].

There are few reported studies on the toxicological effects and signal transduction systems associated with the toxicity of OBs, so there is a marked lack of protocols for their evaluation. Based on their chemical nature and in view of direct contact with them on a daily basis, the need to carry out studies to identify the possible targets of this type of compound. Therefore, this work has two main objectives: first, to identify protein targets for DAST, FB-28, and FB-71 using the reverse docking methodology, and second, to assess their toxicity in *Caenorhabditis elegans* as a preliminary approach to finding possible deleterious effects of these chemicals in nature.

## 2. Materials and Methods

### 2.1. Materials

All in silico calculations were performed using a Dell precision T7600 Minitower workstation with two Intel^®^Xeon^®^ processors CPU E5-268W 0, 3.1 GHz, 8 GB RAM running on the Red Hat Enterprise Linux 5 operating system. The optical brightener (OB) compounds DAST (462268 Sigma-Aldrich, EE.UU., St. Louis, MO, USA), Fluorescence brightener #28 (F3543 Sigma-Aldrich, EE.UU., St. Louis, MO, USA), and Fluorescence brightener #71 (Toronto Research Chemicals, Toronto, ON, Canada) were purchased from the indicated companies. The stock solution for each OB (1000 µM) and subsequent dilutions were prepared using K-medium (KCl, NaCl, Millli-Q Water, Darmstadt, Germany) [23].

### 2.2. Molecular Docking of OBs

#### 2.2.1. Optical Brighteners Structure

**Figure 1:**
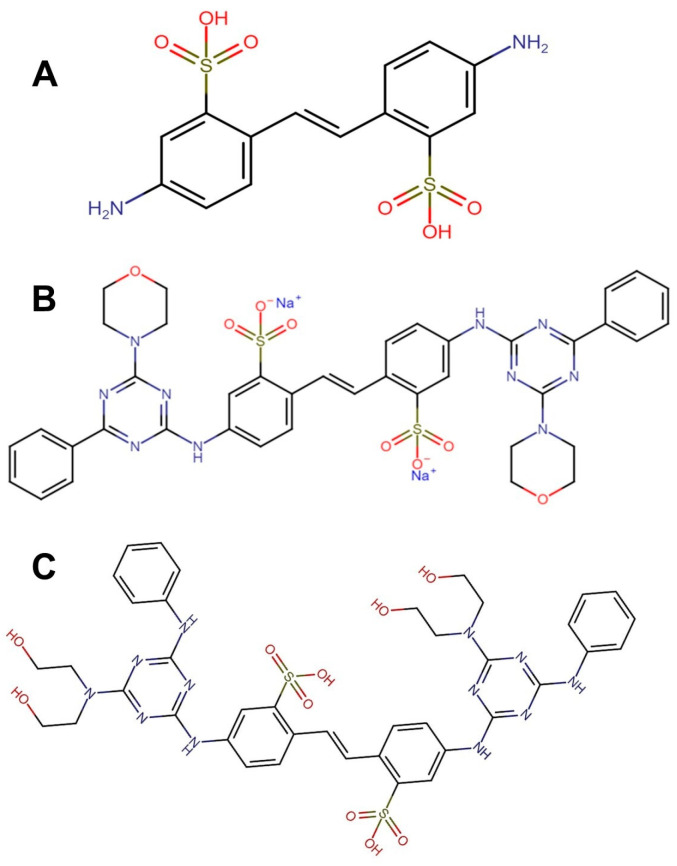
Chemical structure optical brighteners: DAST structure (**A**); FB-71 structure (**B**): FB-28 structure (**C**). The structures were drawn using chEMBL free access (https://www.ebi.ac.uk/chembl/, accessed on 4 March 2023).

The structures of the optical brightness enhancers (Figure 1) were constructed using the Gauss View 5.08 [24] program and were optimized using the density functional theory (DFT) with the B3LYP functional [25] and the 6-311G basis set in the Gaussian 03 program. Following optimization, the output file was converted to .pdb with the program Open Babel and .pdbqt with the AutoDock Tools program (Version 4.2.6) [26].

#### 2.2.2. Preparation of Protein Structures

Protein selection was performed using the PharmMapper web server (http://59.78.96.61/pharmmapper/, accessed on 1 February 2020) and the Environmental and Computational Chemistry Group (ECCG) database. The PharmMapper platform allows us to find target proteins for a particular ligand using a probing process on potential binding sites to proteins stored in the PharmTargetDB database. For this purpose, the server locates the aligned ligand in front of each of the proteins present in the database and then, through algorithms, generates the optimization of the protein–ligand pair, yielding the data of the best energy-based interaction adjustments of affinity and spatial disposition [27]. The PharmMapper output allowed us to obtain those human proteins contained in PharmTargetDB with the 25 best “score” scores for each OB (Table S1). The protein structures selected by PharmMapper were downloaded from the Protein Data Bank database, PDB (http://www.rcsb.org/pdb/home/, accessed on 1 June 2020) in pdb format. In addition to these proteins, a total of 844 proteins from our group were also tested (Table S2).

Prior to the process of coupling with the OBs, these proteins were opened with the program Sybyl-X 8.1 and prepared using the tool of preparation of structures that the program contains. All ions, water molecules and other substructures were removed from each protein prior to docking. Protein structures were pre-analyzed and prepared for docking runs using the biopolymer structure preparation tool with adjustments as implemented in the Sybyl program. The resulting geometry was subsequently optimized via the Powell method using the Kollman United force field, AMBER load, dielectric constant 1.0, NB cutoff 8.0, maximal interactions 100, and a termination gradient of 0.001 kcal/mol.

The minimized pdb-format file was read directly in MGL Tools/AutoDock Tools, which was used to prepare the input .pdbqt files required by AutoDock Vina to obtain the data set and size of the grid box. Kollman charges and polar hydrogen atoms were added to the three-dimensional structure of the proteins using this software, and then the AutoDock Tools program was used to obtain each protein in the pdbqt extension file and to add the polar hydrogens for the docking calculations [28].

#### 2.2.3. Virtual Screening Protocol

The docking coordinates were determined through a grid box in AutoDock Tools using a blind docking strategy with a spacing of 1.0 Å and centered in the macromolecule to include all possible link sites for each OB [29,30]. The docking study was carried out using AutoDock Vina 1.0, and a virtual screening program run on Linux employed a number of modes equal to 20 and an exhaustiveness of 20. The coupling affinity results were used to classify the white proteins of the OBs.

#### 2.2.4. Refinement Docking Experiments

The docking refinement experiments with 100-run repetitions were performed with the proteins having the highest affinity values (less than −9 kcal/mol) for better precision in the results. This was achieved by performing the blind docking as a strategy in AutoDock Vina [31]. The parameters for this step were a number of modes of 50 and exhaustiveness of 100. Each docking calculation was performed in triplicate for each OB.

#### 2.2.5. Conformational Analysis

The LigandScout 3.0 program was used to determine the existing interactions [32] for protein–OB complexes with the highest affinity values. The interaction cutoff threshold of the PDB interpretation settings was set to 7.0 Å. This threshold defines a sphere (in Å) around the ligand. The atoms of the protein enclosed inside the sphere were considered to be possibly involved in the interactions. All of the remaining settings were maintained as the default [33]. The validation of this method has been reported in other articles [34,35].

#### 2.2.6. Molecular Dynamics Simulations

The proteins that presented the best binding affinity with DAST, FB-71 and FB-28 (Table 1) were subjected to molecular dynamics simulations (MDS) with the objective of evaluating the stability of the complexes formed and corroborating the energetic calculations. An MDS of 100 ns was performed using Gromacs version 2020.2 [36]. The force fields used for the protein and the ligand were the CHARMM force field [37] and the CHARMM General Force Field (CGenFF) [38], respectively. For a benchmark, simulations were also performed for the selected proteins with their respective cocrystalized ligands.

All complexes were immersed in a cubic periodic box, in which each complex was solvated with TIP3P water under periodic boundary conditions [39]. The systems were neutralized, and the ionic strength (0.1 mol L^−^^1^) of the medium was adjusted by adding Na^+^ and Cl^−^ ions, keeping the number of particles constant. Energy minimization of the systems was performed until the energy converged. Next, an equilibrium phase was carried out keeping the pressure and temperature (NVT and NPT ensemble) constant at 300 K and 1.0 bar, and the equilibration periods were 1.0 ns. The production runs were 100 ns, and the trajectories were saved every 0.01 ns. The data obtained from the MDS were used as inputs to calculate the root mean square deviation (RMSD), and the root mean square fluctuation (RMSF) and radius of gyration (Rg) were determined to analyze the flexibility and stability of the complex over time. The MMPBSA.py script [40] in the AMBER 21 suite was used to predict the free binding energies of these protein–ligand complexes. To apply the MMPBSA.py script, the topologic file, coordinates file, and production file generated in Gromacs were converted to their counterparts in Amber. The interaction energy and solvation free energy for the complex, receptor, and ligand were calculated, and the results were averaged to obtain an estimate of the binding free energy according to the MM/GBSA approaches [41].

Research works in which an experimental work is carried out with a previous theoretical result have been of great help in understanding how certain compounds that are used on a daily basis could affect, for example, the biological processes that occur in the human body. In order to corroborate the results obtained from docking, experimental tests were carried out to evaluate the toxicity of OBs using *C. elegans* nematodes as a biological model.

### 2.3. Toxicity of OBs in Experiments with Caenorhabditis elegans

#### 2.3.1. Optical Brightener

The optical brightener (OB) compounds DAST (462268 Aldrich, St. Louis, MO, USA), Fluorescence brightener #28 (FB-28, F3543 Sigma, St. Louis, MO, USA), and Fluorescence brightener #71 (FB-71, Toronto Research Chemicals, Toronto, ON, Canada) were purchased from the indicated companies and dissolved in K-medium (KCl, NaCl, Milli-Q Water, Darmstadt, Germany) at 1000 µM concentration as a stock solution and diluted when used.

#### 2.3.2. Strains and Nematodes: Maintenance and Treatments

The *Caenorhabditis elegans* N2 strains (wild type) and transgenic nematodes (*gpx-4*::GFP (BC20305), *gpx-6*::GFP (BC17553), *sod-4*::GFP (BC20333), *hsp-4*::GFP (SJ4005), and *gst-1*::GFP (BC20316)) were obtained from the Caenorhabditis Genetics Center (CGC) (University of Minnesota, Minneapolis, MN 55455, USA).

The worms were maintained on nematode growth medium (NGM) at 20 °C [42] and *Escherichia coli*-OP50 as a food source, as suggested in the literature [43]. Nematode larvae were synchronized by bleaching (NaClO, NaOH beads, and Milli-Q water)—which destroyed the worms, but eggs were protected by their shell—followed by several washes with K-medium. Experiments were conducted with the same larval stage to guarantee reproducibility [44].

#### 2.3.3. Lethality Assay

The lethality assay was performed using K-medium in 96-well microplates. Nematodes (10 ± 2 for treatment) of larval age L4 were exposed for 24 h to OBs (DAST, FB-28 and FB-71) at 100, 250, 500, 750, 1000, and 5000 µM concentrations. After 24 h or 48 h, the numbers of live and dead worms were counted through visual inspection using a microscope. All experiments detailed above were carried out three times, with three replicates per treatment used for each new stock solution (1 mM) [45].

#### 2.3.4. Body Length Assay

Nematode larvae (L1) were exposed to different OB concentrations and grown at 20 °C for 48 h in K-medium. Attenuated *E. coli* (OP50) was employed as a food source. A Nikon SMZ 745T dissection microscope was used to measure body length by capturing images of the nematode. Images were then processed using ImageJ software (Version 1.53) [46]. Approximately 30 nematodes were randomly measured for each replicate across three independent experiments.

#### 2.3.5. Locomotion Assay

After 24 h of exposure to different OB concentrations at 20 °C, the nematodes in stage L4 (10 ± 2 for treatment) were transferred to a plate with agar and scored for the number of body bends in 20 s, considering the individual number of forward sinusoidal movements. All experiments detailed above were carried out three times, with three replicates per treatment [47].

#### 2.3.6. Reproduction Assay

Age-synchronized N2 (L4 stage) were exposed to the different treatment concentrations for 24 h. Subsequently, five worms from each treatment were placed in a Petri dish containing only food and allowed to deposit embryos. Egg counting was carried out once laying started every day for 72 h at 20 °C [47].

#### 2.3.7. Gene Expression Using Fluorescence Measuring

The effects on gene expression were monitored utilizing GFP transgenic *C. elegans* strains containing the *gst-1*, *hsp-3*, *hsp-4*, *sod-4,* and *gpx-4* genes. The stress-reporter strains were grown at 20 °C and seeded with *E. coli* (strain OP50). Synchronized worms (≈350 nematodes for treatment) were exposed to different OB concentrations less than the LC_50_ (50, 100, 150, 250, and 500 µM) and placed into black nonfluorescent U-bottomed 96-well microplates for 24 h. Fluorescence readings were performed with a Varioskan™ LUX multimode microplate reader at 485/525 nm excitation/emission wavelengths, respectively. K-medium was used as a control. Three replicates were used per treatment, and the experiment was repeated three times. Data were normalized against the vehicle control (K-medium) [45].

### 2.4. Statistical Analysis

Data are reported as mean ± standard error of the mean (SEM). The homogeneity of the variance and the normality were verified using the Kolmogorov–Smirnov tests. Significant differences between means were determined with ANOVA one-way, using the Dunnett test as a post-test, comparing each treatment with the control. Statistical significance was set at *p* < 0.05. The graphs were generated using GraphPad Prism 8.0 (GraphPad Prism Software, Inc., San Diego, CA, USA).

## 3. Results

### 3.1. Protein Target Selection

One of the fundamental methods in research for identifying potentially favorable affinity-based protein–ligand interactions is molecular docking [48]. The output from PharmMapper for both OBs consisted of a total of 300 human proteins (the output allowed us to obtain those human proteins contained in PharmTargetDB with the 25 best “score” for each OB (Table S1)), and from the use of ECGG, a total of 840 proteins were evaluated (Table S2). The best results obtained from both databases are shown in Table 1.

Taking into account the two databases used in this research, a total of 844 proteins were used to perform the inverse docking calculations with each OB evaluated, the 25 best binding energy values for each OB are shown in Table 1. The energy values in the complexes generated with DAST oscillate between −9.6 and −11.2 kcal/mol; values with FB-28 oscillate between −11.0 and −13.3 kcal/mol and values with FB-71 oscillate between −13.4 and −15.3 kcal/mol.

The best docking affinity values (lower than −10.0 kcal/mol) for both OBs were found for the complexes Hsp70-1/DAST, with an affinity score of −11.2 kcal/mol; the CD40 ligand/FB-71, with an affinity score of −15.3 kcal/mol; and the CD40 ligand/FB-28, with an affinity score of −13.3 kcal/mol. For OB FB-71, better results were obtained compared to DAST and FB-28; in the latter, for DAST, only a total of 11 proteins were obtained with affinity values lower than −10.0 kcal/mol.

The proteins with PDB ID: 3LKJ, 4IDT, 3EAH, 1NSI, 3UA1, and 3O96 were common in all OBs analyzed, with the CD40 ligand presenting affinity values below −10.0 kcal/mol for all OBs because this protein was recently reported to inhibit receptor binding and function of the constitutively trimeric tumor necrosis factor (TNF) family cytokine CD40 ligand [49,50]; mitogen-activated protein kinase 14 (NIK) had a similar behavior. The proteins with PDB ID: 3W3J, 3CO6, 2VQQ, 2OC2, 1O8A, and 1LYW were common between FB-71 and FB-28 OBs, and the 4EY7 protein was common between DAST and FB-28.

Therefore, the best complex for each OB was subjected to molecular dynamics simulations to further deepen our knowledge about their interaction profiles and to examine their conformational stability over a period of time.

### 3.2. Conformational Analysis

**Figure 2:**
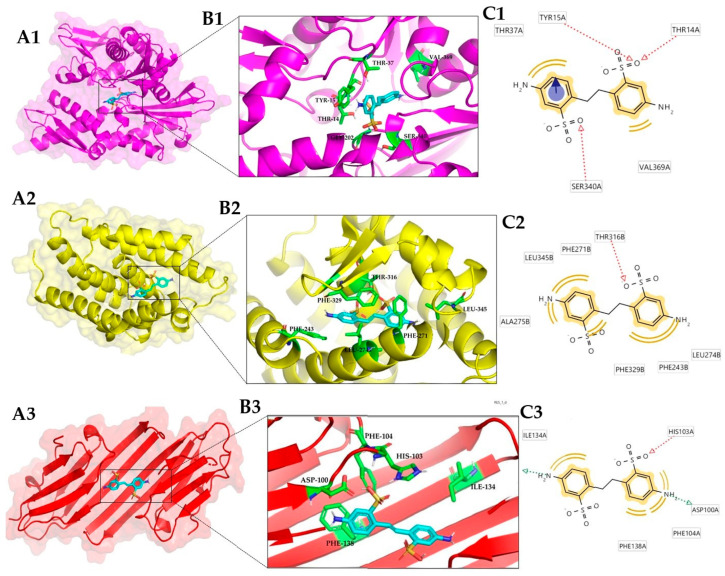
Three-dimensional view of the three best complexes with DAST: Hsp70-1 (3JXU) (1); LXR-beta receptor (1P8D) (2) and Hsp27 (4MJH) (3). Protein–ligand complex (**A**); binding site (**B**); and ligand–residue interactions (two-dimensional view) (**C**).

The results from LigandScout 4.1 show that the more commonly found interactions in protein/OB complexes correspond to hydrophobic, as well as interaction ring aromatic, hydrogen bond donor and bond acceptor types. Using an interaction cutoff threshold of 7 Å in LigandScout 3.0 [32]. The predicted interactions in DAST/protein complexes are hydrophobic and hydrogen bond donor interactions, and the contact residues participating in greater proportions are threonine and phenylalanine. The contact residues participating in the interaction with DAST are mostly threonine, Thr14a, and Thr37a for Hsp70-1 (Figure 2(A1–C1)), presenting hydrogen bond donor interactions; phenylalanine, Phe243b, Phe271b, and Phe329b for LXR-beta receptor (Figure 2(A2–C2)); and Phe104a and Phe138a for Hsp27 (Figure 2(A3–C3)), presenting hydrophobic interactions.

**Figure 3:**
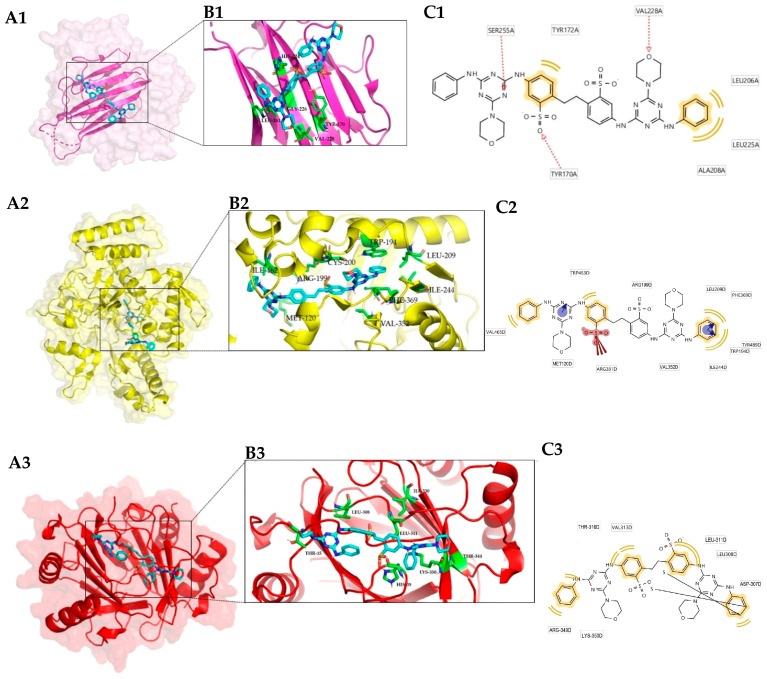
Three-dimensional view of the three best complexes with FB-71: CD40 ligand (3LKJ) (1); nitric oxide synthase (1NSI) (2) and platelet factor 4 (1F9Q) (3). Protein–ligand complex (**A**); binding site (**B**); and interaction with residues in the binding site (two-dimensional view) (**C**).

In the best complex formed with FB-71, the predicted interactions were hydrophobic interactions (Figure 3) involving mostly valine, tyrosine, and leucine residues. In the case of the CD40 ligand/FB-71 complex (Figure 3(A1–C1)), the residues involved in this type of interaction were Leu168a, Tyr170a, His224a, Gly226a, Val228a, and Leu261a.

In the interactions present in the Nitric oxide synthase/FB-71 complex (Figure 3(A2–C2)), the residues involved were Met120d (hydrogen bond donor), Trp194d, Leu209d, Ile244d, Val352d, Phe369d (hydrophobic interaction), Gln387d (hydrogen bond donor), and Tyr489d (hydrogen bond acceptor). The prevailing hydrophobic interactions similar to those in the CD40 ligand/FB-71 complex and the contact residues that participated in the interaction between Platelet factor 4 and FB-71 (Figure 3(A3–C3)) were Leu308d, Leu311d, Ile330d, Thr344d, and Lys350d, which are all hydrophobic interactions.

**Figure 4:**
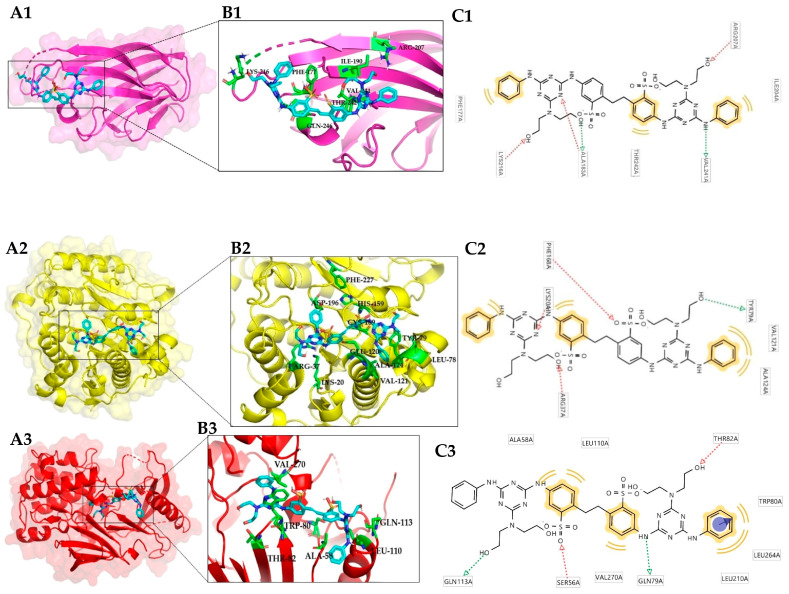
Three-dimensional view of the three best complexes with FB-28: CD40 ligand (3LKJ) (1); histone deacetylase 4 (2VQQ) (2) and RAC-alpha serine/threonine-protein kinase (3O96) (3). Protein–ligand complex (**A**); binding site (**B**); and interaction with residues in the binding site (two-dimensional view) (**C**).

In the case of the complexes formed with FB-28, the best complex was formed with the CD40 ligand. This protein also formed the best complex with FB-71. Even when the binding site was different in both complexes (Figure 3(A1) and Figure 4(A1)) the stability of the protein was preserved, and the energy values were below 10 kcal/mol. In the case of the CD40 ligand/FB-28 complex, hydrophobic interactions can be observed with the residues ALA-183a, THR-242a, ILE-204a and hydrogen bonds with the residues VAL-241a, PHE-177a, LYS-216a, and ARG-207a.

### 3.3. Molecular Dynamics Simulation

Molecular dynamics simulations of DAST with the Hsp70-1 protein, and the ligands FB-71 and FB-28 with the CD40-L protein, were performed during a running time of 100 ns to analyze the flexibility and stability of each complex over time. Next, the stability of Hsp70-1 and CD40-L with their respective cocrystallized ligands was also simulated under the same conditions to compare the existing conformational and energetic changes through calculations of RMSD, RMSF, Rg, and MMGBSA.

#### 3.3.1. Root Mean Square Deviations (RSMD)

**Figure 5:**
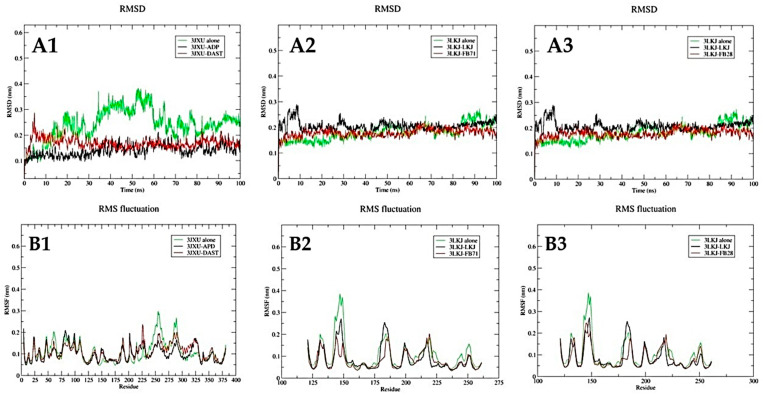
Backbone RMSD plots of the Hsp70-1 (3JXU) protein alone, with native ligand (ADP) and DAST (**A1**); CD40-L (3LKJ) protein alone, with native ligand (LKJ) and FB-71 (**A2**); CD40-L (3LKJ) protein alone, with native ligand (LKJ) and FB-28 (**A3**); and RMSF plots of the Hsp70-1 protein alone, with native ligand (ADP) and DAST (**B1**); CD40-L protein alone, with native ligand (LKJ) and FB-71 (**B2**); and CD40-L protein alone, with native ligand (LKJ) and FB-28 (**B3**).

When carrying out a stability analysis of a ligand-protein complex, one of the important aspects to take into account is the RMSD, and low values of this parameter are obtained, which is an indication that the ligand–protein system is balanced and stable. The RMSD was calculated for the Cα atoms of the Hsp70-1 protein backbone with the DAST ligand, the CD40-L protein with the FB-71 ligand, and the CD40-L protein with the FB-28 ligand as well as with their cocrystalized ligands. In addition, the RMSD were also calculated for the proteins without ligands. The RMSD results obtained are shown graphically in Figure 5.

**Figure 6:**
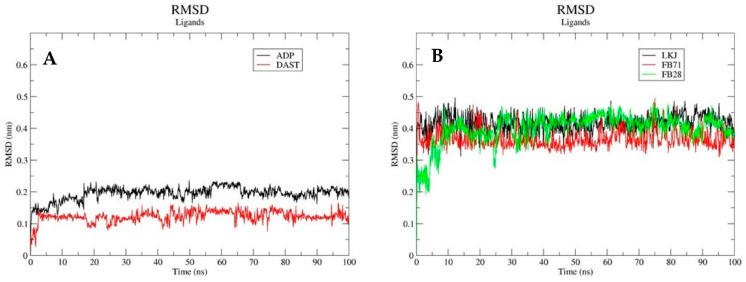
Backbone RMSD plots of ligands. Native ligand (ADP) and DAST (**A**) and native ligand (LKJ), FB-71, and FB-28 (**B**).

The RMSD results show that the backbone atoms of Hsp70-1 in complex with the cocrystalized ligand (ADP) and DAST undergo minimal conformational changes in their conformation. The minimum and maximum RMSD obtained for Hsp70-1-ADP were 0.1 and 0.2 nm, respectively, and those for Hsp70-1-DAST were 0.05 and 0.28 nm, respectively. The first 30 ns for the Hsp70-1-dast complex present fluctuations that vary between 0.1 and 0.25 nm, and after 30 ns, an equilibrium is reached that is very similar to that exhibited by the Hsp70-1-ADP complex (Figure 5(A1)). The RMSD values for the protein alone vary between 0.1 nm and 0.4 nm. In the RSMD graph, it can be seen that the variations are more noticeable when the protein is without a ligand (Figure 5(A1)). In Figure 6A, you can see the RMSD graph for the native ligand (ADP) and for DAST. The RMSD values range between 0.12 nm and 0.14 nm for DAST, while for ADP, they range between 0.15 nm and 0.2 nm. These low values of RMSD suggest that the ligands that interact with 3JXU do not undergo abrupt changes in their position throughout the simulation. In Figure 6B, you can see the RMSD graph for the native ligand (LKJ) and for the two external ligands (FB71 and FB28) that interact with 3LKJ. The RMSD values for the three ligands range between 0.35 nm and 0.45 nm, maintaining these values throughout the simulation, which indicates that they do not suffer abrupt changes throughout the simulation.

In the case of the complexes analyzed for CD40-L with its cocrystallized ligand (LKJ) and FB-71 ligand, Figure 5(A2) shows the RMSD values for both complexes, where the minimum and maximum RMSD values obtained for CD40-LKJ were 0.15 and 0.29 nm, respectively, and those for CD40-FB-71 were 0.05 and 0.22 nm, respectively. The first 10 ns of the CD40-L-LKJ complex present fluctuations that vary between 0.2 and 0.28 nm, and after 10 ns, equilibrium is reached. On the other hand, the CD40-L-LKJ complex presents average fluctuations of 0.15 nm during the entire simulation (Figure 5(A2)). In the case of this same protein with the FB-28 ligand (Figure 5(A3)), the RMSD values were lower than those obtained with the co-crystallized ligand, presenting fluctuations between 0.14 and 0.18 nm, showing stability between 0 and 30 ns and later after 70 ns. In general, the low RMSD values present in the Hsp70-1-DAST, CD40-L-FB-71, and CD40-L-FB-28 complexes suggest that the complexes are stable over time. In the case of the CD40 protein without the ligand, it can be seen that it presents very small RMSD variations (see Figure 5(A2,A3)).

#### 3.3.2. Root Mean Square Fluctuations (RSMF)

One way in which the conformational stability of a protein-ligand complex can also be analyzed is by taking into account the mobility of the residues and calculating the RMSF parameter, which analyzes the mobility of all the amino acids of the protein during the simulation time. In the analysis of the RMSF profile of Hsp70-1 with DAST and its cocrystallized ligand (ADP), it can be observed that they present similar behavior. With DAST, the largest fluctuations were approximately 0.16 and 0.22 nm between amino acids 225 and 228, indicating that in these residues, there is greater flexibility. In the case of ADP, the fluctuation was approximately 0.16 and 0.20 nm between amino acids 77 and 85 (Figure 5(B1)). When the RMSF profile of Hsp70-1 alone is analyzed, a fluctuation between amino acids 235 and 275 is identified. This is because Hsp70-1 bound to ADP presents several important interactions in this region with residues Gly 339, Glu 268, Lys 271, and Ser 273. In the case of Hsp70-1 linked to DAST, an interaction with Ser 340 and with Val 369 can be seen in Figure 2(C1).

On the other hand, in the analysis of the RMSF profile of CD40-L with FB-71 and its cocrystallized ligand (LKJ), both ligands behaved similarly; however, significant fluctuations were observed in amino acids ~140–150 and ~180–190. This flexibility may be related to the beta-turn motif present in these stretches of the CD40-L secondary structure. With LKJ, the largest fluctuations were approximately 0.20 and 0.28 nm between amino acids 146 and 148, while in that same region for FB-71, the RMSD values oscillated between 0.09 and 0.15 nm, indicating that in these residues, there is greater flexibility. A similar behavior is observed between amino acids 183 and 184 (Figure 5(B2)). In the analysis of this same protein with the FB-28 ligand, both ligands behaved similarly; however, significant fluctuations were observed in amino acids ~175–190 (Figure 5(B3)), for which the LKJ ligand presents a fluctuation. It is possible that some interaction between the amino acids of that region of the protein occurs with FB-71 and FB-28, but such interaction does not occur with the LKJ ligand. When the RMSF profile of CD40-L alone is analyzed, it is possible to identify a fluctuation between amino acids 140 and 150. This is because CD40-L bound to LKJ presents an interaction with Tyr 145.

#### 3.3.3. Radius of Gyration (Rg)

The radius of gyration (Rg) was determined for the Hsp70-1 and CD40-L complexes. The radius of gyration is a physical quantity that describes the compactness of the protein structure, and lower values of Rg describe a more rigid structure during the simulation.

**Figure 7:**
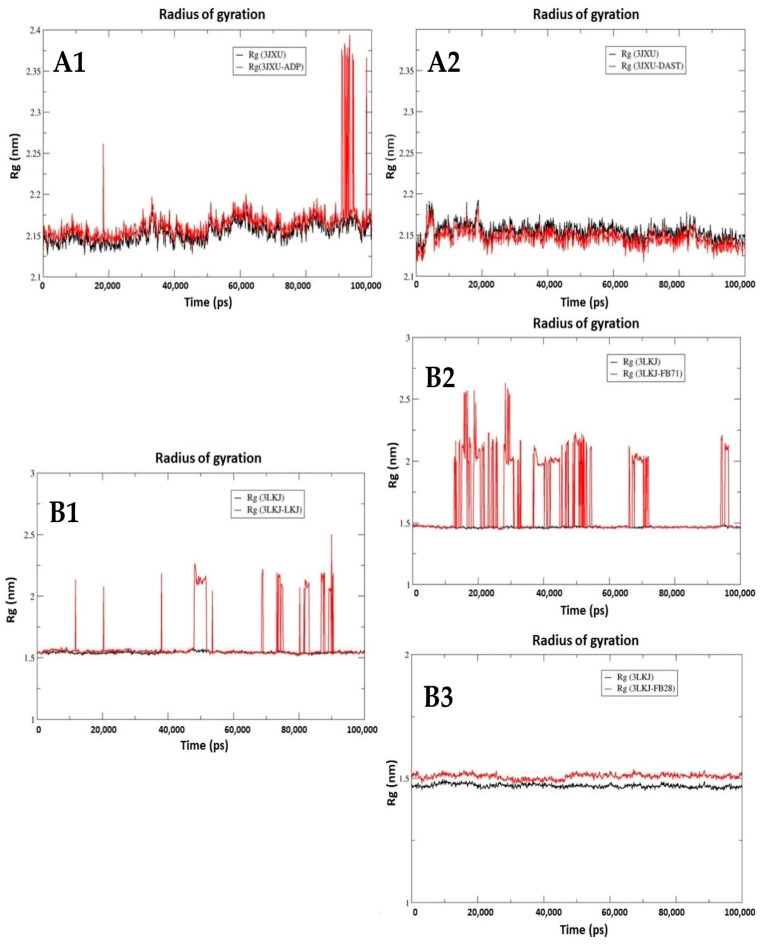
Time evolution of radius of gyration (Rg) of the Hsp70-1 (3JXU) protein with native ligand (ADP) (**A1**); Hsp70-1 (3JXU) protein with DAST (**A2**); CD40-L (3LKJ) protein with native ligand (LKJ) (**B1**); CD40-L (3LKJ) protein with FB-71 (**B2**); and CD40-L (3LKJ) protein with FB-28 (**B3**).

The Hsp70-1-ADP complex maintained equilibrium with an average Rg value of 2.16 nm in the first 90,000 ps (90 ns). From 90,000 ps to 95,000 ps, there were fluctuations between 2.15 and 2.35 nm (Figure 7(A1)), while Hsp70-1 without ligand maintained an average Rg of 2.14 nm throughout the simulation. For the Hsp70-1-DAST complex, in the first 10,000 ps (10 ns), there were fluctuations between 2.12 and 2.18 nm, and from 10,000 ps to 100,000 ps, equilibrium was maintained with an average Rg value of 2.14 nm. Hsp70-1 without ligand maintained an average Rg of 2.15 nm throughout the simulation, similar to the behavior shown for the Hsp70-1-DAST complex (Figure 7(A2)). The CD40-L-LKJ complex presents fluctuations in the value of Rg throughout the simulation, and these changes are more noticeable after 70,000 ps (70 ns), with values between 1.5 and 2.5 nm (Figure 7(B1)). CD40-L without ligand maintains an average Rg of 1.52 nm throughout the simulation.

The CD40-L-FB-71 complex presents fluctuations in the value of Rg throughout the simulation, and these changes are more between 15,000 and 55,000 ps, with values between 1.5 and 2.5 nm (Figure 7(B2)). CD40-L without ligand maintains an average Rg of 1.5 nm throughout the simulation, and the same behavior is observed for CD40 without ligand with an average Rg of 1.4 nm (Figure 7(B3)).

#### 3.3.4. Molecular Mechanics Energies Combined with Surface Area Continuum Solvation (MMGBSA)

MMGBSA calculations in molecular dynamics allowed estimation of the total binding free energy of Hsp70-1 complexes with ADP and DAST, and CD40-L with LKJ, FB-71, and FB-28 (Table 2). A negative value of the total binding free energy suggests a stable complex, and a positive value suggests an unstable complex. The energy contributions for each ligand are presented below. Values are presented as mean ± SEM, for these energy calculations, and 50 frames were taken throughout the entire simulation. Within the complexes studied, Hsp70-1 had a more favorable binding free energy with ADP (−25.96 kcal/mol) than with the ligand DAST (−10.23 kcal/mol). On the other hand, the CD40-L-LKJ, CD40-L-FB-71, and CD40-L-FB-28 complexes have binding energy values of −17.56, −10.53, and −18.03 kcal/mol, respectively.

The MMGBSA calculations support those reported by the docking calculations, suggesting that the Hsp70-1-DAST, CD40-L-FB-71, and CD40-L-FB-28 complexes are thermodynamically stable.

### 3.4. Toxicity of OBs in Caenorhabditis elegans 

#### 3.4.1. Lethality Assay

**Figure 8:**
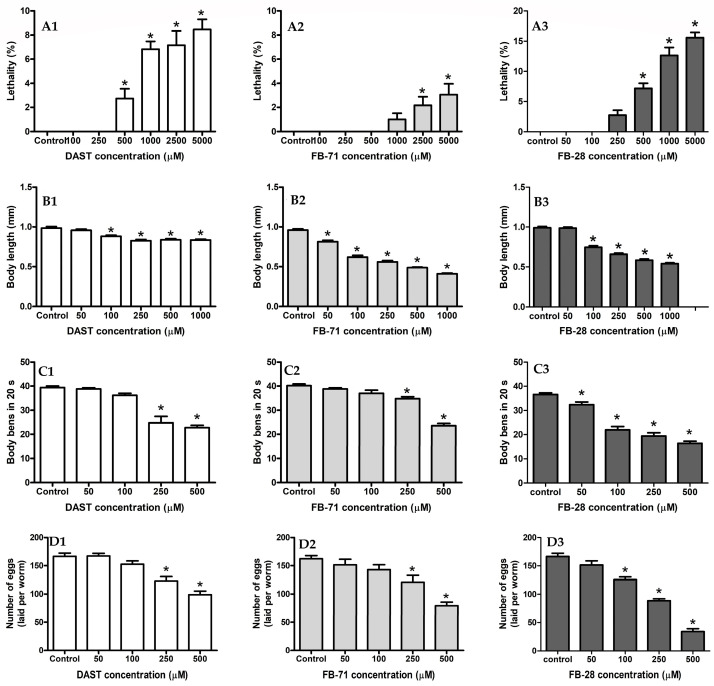
Lethality (**A**), growth (**B**), locomotion (**C**), and reproduction (**D**) effects in *C. elegans* exposed to DAST (1), FB-71 (2), and FB-28 (3). *. Significant difference (*p* < 0.05) compared to control.

The results of the lethality assays showed reduced survival of the L4 larvae after 24 h of exposure to DAST, FB-71, and FB-28 in most samples. The lethality results are shown in Figure 8(A1–A3). At the maximum tested concentrations of DAST, FB-71, and FB-28 (5000 μM), only 8.5, 3.6, and 17.1% lethality values were recorded, respectively.

#### 3.4.2. Body Length Assay

The effects of DAST, FB-71, and FB-28 on the growth of *C. elegans* were assessed and showed significant differences (*p* < 0.05) with respect to the control for both OBs. FB-71 displayed extensive growth inhibition at concentrations smaller than those of DAST and FB-28 (Figure 8(B1–B3)); FB-28 (55%) displayed more extensive growth inhibition than FB-71 (52%) and DAST (19%).

#### 3.4.3. Locomotion Assay

The effect of movement behavior was analyzed and is shown in Figure 8(C1–C3). Both DAST and FB-71 OBs decreased by approximately 43% and FB-28 54% at the highest concentration tested (500 µM) compared to the control, with typical sinusoidal movements in *C. elegans* after 24 h of exposure (Figure 8(C1–C3)). FB-28 was slightly more potent than DAST and FB-71 in inducing locomotion inhibition, especially at 250 µM, with 34, 28, and 15%, respectively.

#### 3.4.4. Reproduction Assay

The results of the effect of DAST, FB-71, and FB-28 on the reproduction of *C. elegans* are shown in Figure 8(D1–D3). FB-28 caused a slightly more significant effect than FB-71 and DAST on the egg-laying capacity of *C. elegans*, reducing it even at the lowest concentration (50 µM), with reductions of 9, 7, and 0.5% relative to the control group, respectively. The maximum reproduction inhibition for each chemical was achieved at 500 µM, resulting in reductions of 79, 51, and 40% in reproductive rates, respectively, compared to the control.

#### 3.4.5. Transgenic Reporter Assays

The evaluation of the relative changes in the gene expression of *C. elegans* exposed to OBs was carried out using different transgenic strains (*gfp*-reporter) associated with cellular stress, oxidative stress, and metabolism activation, and the results are shown in Table 3. Gene expression measurements are displayed as the mean ± standard error of the mean (SEM). For all OBs, the variation in the gene expression of *C. elegans* in the different transgenic strains evaluated occurred following a concentration-response fashion after 24 h of exposure.

Analyzing the results obtained, DAST elicited the highest gene expression response in the following order: *gpx-4* (3.2-fold) > *sod-4* (3-fold) > *gst-1* (2.2-fold) > *hsp-3* (1.5-fold) > *hsp-4* (1.4-fold) with respect to FB-71 that followed the next order: *gpx-4* and *sod-4* same expression (1.7-fold) > *hsp-3* (1.5-fold) > *hsp-4* (1.4-fold) > *gst-1* (1.1-fold); in the case of FB-28, it showed the lowest gene expression response in the following order: *gst-1* and *hsp-4* same expression (1.2-fold) > *hsp-3* and *sod-4* same expression (1.1-fold) > *gpx-4* (no change from control). This effect was obtained at the maximum concentration tested for both OBs (500 µM). Interestingly, DAST and FB-71 increased the expression of the *gpx-4* and *sod-4* genes associated with oxidative stress, indicating a toxic response related to the generation of reactive oxygen species (ROS). In all cases, a clear concentration–response relationship was observed.

## 4. Discussion

Currently, increasing economic resources are being invested in the synthesis and application of OBs due to the versatility of their uses in the textile industry [51]—detergents, agriculture [52], polymers [3,53], among others. However, if we look in the literature for studies about the toxicity that these compounds can generate, especially those derived from stilbene (which make up most of the market), it is limited [54]. Additionally, given the chemical nature of these compounds, they are not easily degraded naturally by sunlight, and even when using water treatment methods, only partial degradation can be achieved, which could have an adverse effect on aquatic systems [54,55].

That is why this study focused on the evaluation of the toxicological profile of three optical brighteners from the theoretical point of view and in vivo using *C. elegans* as a biological model, which has been widely characterized and used for the evaluation of toxicological profiles of different compounds.

### 4.1. In Silico Behavior of OBs

The results obtained from virtual screening were carried out through docking protocols for DAST, FB-71, and FB-28, which show high affinities for some target proteins. We can highlight proteins involved in glutathione metabolism, apoptosis, the insulin pathway, and phospholipid metabolism, among others. These results suggest that OBs have the potential to alter the mechanisms involved in cell death.

These molecular docking simulations demonstrate that the best complex obtained for DAST was Hsp70-1/DAST. DAST interacts with residues Thr14 and Thr15 through hydrophobic interactions, which have been reported to be part of the active site of this protein. 3JXU (heat shock protein 1A (*Hsp70-1*)) is a chaperone that plays a key role in the protein quality control system [56] by maintaining protein homeostasis during cellular (oxidative) stress through two opposing mechanisms: protein refolding and degradation [57]. This is consistent with the results obtained in the trials with transgenic GFP-reporter strains of *C. elegans*, in which DAST presented the highest gene expression with the strains associated with cellular stress (*hsp-3* and *hsp-4*) and oxidative stress (*gpx-4* and *sod-4*).

In the interaction of the LXR-beta receptor/DAST complex, an important residue, Phe239, was found, which also participates in the interaction of a synthetic inhibitor (CO1) of this protein. Interactions with the Thr316 residue have also been reported as possible interactions that could cause LXR-beta receptor inhibition [58]. These nuclear receptor families exhibit functions as transcriptional regulators of lipid metabolism that appear to control inflammatory processes [58,59]. A similar behavior was presented in the Hsp27/DAST complex, where the four residues (Phe104, Phe138, Asp100, and His103) with which it presented a greater interaction with the DAST ligand have been reported to be part of the active site of this protein, which is part of the *HSP* family and plays an important role in the development of multiple diseases associated with oxidative stress.

A great number of complexes were found for FB-71, and a better affinity value was obtained for the complex formed with CD40 ligand (CD40L ligand). A member of the tumor necrosis factor (TNF) family cytokine that plays an essential role in humoral and cellular immune responses, a soluble form has been reported to express biological activities similar to the transmembrane form [49,50]. The second-best complex for FB-71 was with inducible nitric oxide synthase (NOS-2) (1NSI).

The inducible type of nitric oxide synthase (NOS) is a family of enzymes that produce nitric oxide (NO) from L-arginine in the presence of NADPH and O_2_. Nitric oxide (NO) is an important mediator of inflammatory responses in the lung, plays a role in cell proliferation and differentiation and in the protection of oxidative stress, and is considered to be a key molecule in the immune responses to bacteria, parasites, and tumors. Some studies have linked an inhibition of NOS-2 to high concentrations of NO, affecting reproductive functions. It has been reported that it causes erectile dysfunction in men, and ectopic pregnancies and low fertility in women [60,61]. This could be evidenced in the results obtained with the biological model *C. elegans* exposed to OBs, showing an inhibition in the egg-laying capacity. The affinity energy values of the complexes formed with FB-28 also showed values above those of DAST, obtaining the following affinity behavior: FB-71 > FB-28 > DAST. The best complex formed by FB-28 was also with a CD40 ligand (CD40L ligand), and although FB-28 bound in a different site of the protein with respect to FB-71, the stability of the protein was maintained, which can be evidenced by the results obtained from RMSD and RMSF. The second-best complex with FB-28 was with HDAC4 (2VQQ), which plays an important role in transcriptional regulation, cell cycle progression and developmental events; it is also involved in muscle maturation through its interaction with MEF2A, MEF2C, and MEF2D.

### 4.2. Effects of OBs on the Physiology and Gene Expression of C. elegans

The OBs tested in this study—DAS, FB-71, and FB-28—showed a low lethality rate in *C. elegans*, and this behavior has also been reported in studies of OBs with similar chemical structures [62]. On the other hand, the effects on the inhibition of growth, locomotion and reproduction of *C. elegans* nematodes exposed to DAST, FB-71, and FB-28 OBs are more noticeable with respect to lethality. This could be attributed to the oxidative stress that OBs can induce in *C. elegans,* giving rise to the generation of reactive oxygen species (ROS), which do not cause a lethal effect on *C*. *elegans* but cause severe changes in physiological aspects, including reduced movement, a limited rate of progeny production, and downregulation of growth. This is consistent with the results obtained in the tests of transgenic strains where the genes that caused the highest expression were those associated with oxidative stress *gpx*-*4* and *sod*-*4* for both OBs, from which it could be inferred that all these changes in the physiology of *C*. *elegans* are due to the accumulation of ROS at the intracellular level. Studies of commercial OBs derived from stilbene, such as those tested in the present study, have shown an effect on the reduction of respiratory rate in microorganisms, generating incomplete electron transfer within the respiratory chain, which leads to the formation of ROS. In addition, a similar structure search was carried out in PubChem (https://pubchem.ncbi.nlm.nih.gov/, accessed on 1 November 2022), and benzenedisulfonic acid was taken as a substructure in order of relevance, which has been reported to cause reproductive damage in rats and frogs, causing malformations in fetuses and inhibition of cell division, respectively, at concentrations of approximately 50 ppm [63,64].

The alteration of gene expression, such as *gst-1* (metabolic gene), has been reported to have a potential effect on the biological and physiological function in *C. elegans* [65], which is consistent with the results obtained in aspects of the physiology of *C. elegans* exposed to both OBs. In addition, heat shock proteins (HSPs), which are stress-sensitive molecular chaperones, are critical for proteome stability, and a variation in these proteins can interfere with the stability of protein folding [66]. Recent studies have shown the important role of HSPs in the degradation of misfolded proteins in neurodegenerative diseases, such as Parkinson’s disease. Increased HSP expression is indicative of neurotoxicity associated with oxidative stress. Both OBs induced an increase in the expression of *hsp-3* and *hsp-4* (which are part of the HSP family), which indicates an attempt at *C. elegans* to protect itself from the toxic and pro-oxidative effects that could be caused by DAST, FB-71, and FB-28 [67]. In addition, studies carried out in *C. elegans* have shown that the *hsp-4* gene under equilibrium conditions is almost undetectable and is only activated under stress [68,69]. This result could imply that exposure of *C. elegans* to DAST, FB-71, and FB-28 affects the proper functioning of ACh and GABA, which are the main neurotransmitters in the motor circuit that function both at motor neurons and body wall muscles, causing an inhibition of locomotion—as recorded in this study—in which both OBs induced a reduction in locomotion by approximately 43% compared to the control [70,71]. In general, the end points in *Caenorhabditis elegans* locomotion, growth, and reproduction assays demonstrated a sensitive and rapid method of assessing the environmental toxicity of OBs.

## 5. Conclusions

The evaluated OBs DAST, FB-71, and FB-28, despite inducing low lethality, showed a significant deleterious effect on *C. elegans* physiology, such as locomotion, growth, and reproduction. The transgenic strains associated with oxidative stress gpx-4 and sod-4 showed the greatest variation with respect to the control for both OBs, which is consistent with the results obtained from docking. Molecular docking showed that DAST and FB71 are possible molecular targets of proteins associated with oxidative stress, inflammatory processes and erectile dysfunction. These results are corroborated by the calculations of RMSD, RMSF, and MMGBSA obtained from the molecular dynamics simulations. Through the results of this study, it is necessary to elucidate the toxic effects of OBs and establish toxicity thresholds, as reference doses, for a precise evaluation of exposure risks using additional biological models to be able to describe the signal transduction systems associated with the toxicity of these compounds, for which there is little literature and therefore little regulation, taking into account the large consumption of optical brightening agents used as products for daily use worldwide. As the use of OBs increases, their different sources—such as detergents, textiles, plastics and paper products—must also be investigated to characterize the primary emissions of OBs to the environment and to develop an adequate regulatory framework.

## Figures and Tables

**Table 1:** The 25 best results from docking with AutoDock Vina (kcal/mol).

DAST			FB-71			FB-28		
PDB ID	Description	Average Affinity Values * (kcal/mol)	PDB ID	Description	Average Affinity Values (kcal/mol)	PDB ID	Description	Average Affinity Values (kcal/mol)
**3JXU**	Heat shock 70 kDa protein 1A	−11.2	**3LKJ**	TNF Family Cytokine CD40 ligand	−15.3	**3LKJ**	TNF Family Cytokine CD40 ligand	−13.3
**1P8D**	Oxysterols receptor LXR-beta	−10.3	**1NSI**	Nitric oxide synthase, inducible	−14.5	**2VQQ**	Histone deacetylase 4	−12.6
**4MJH**	Heat shock protein beta-1 (Hsp27)	−10.3	**1F9Q**	Platelet factor 4	−14.5	**3O96**	RAC-alpha serine/threonine-protein kinase	−11.9
**3LKJ**	TNF Family Cyokine CD40 ligand	−10.2	**1PL1**	glutathione transferase (GST) A1-1	−14.4	**1LYW**	Cathepsin D	−11.9
**4EY7**	Acetylcholinesterase	−10.2	**2OC2**	Angiotensin-converting enzyme	−14.4	**1O8A**	Angiotensin-converting enzyme	−11.8
**1S3X**	Heat shock 70 kDa protein 1A ATPase domain	−10.2	**2VQQ**	Histone deacetylase 4	−14.4	**3EAH**	Nitric oxide synthase, endothelial	−11.7
**4XRY**	Human Cytochrome P450 2D6	−10.2	**1O8A**	Angiotensin-converting enzyme	−14.3	**3CO6**	Forkhead box protein O1	−11.7
**4EY6**	Acetylcholinesterase	−10.1	**3E7G**	Nitric oxide synthase, inducible	−14.2	**4QTD**	Mitogen-activated protein kinase 8	−11.7
**2XSI**	Human serum albumin	−10.1	**5GPG**	Peptidyl-prolyl cis-trans isomerase FKBP3	−14.0	**4D75**	Cytochrome P450 3A4	−11.6
**1RY8**	Prostaglandin F synthase	−10.0	**1LYW**	Cathepsin D	−14.0	**1NSI**	Nitric oxide synthase, inducible	−11.6
**4IDT**	Mitogen-activated protein kinase kinase kinase 14	−10.0	**3CO6**	Forkhead box protein O1	−13.8	**1Z57**	Dual specificity protein kinase CLK1	−11.5
**4NRE**	UGGGGU quadruplexes	−9.8	**3LN1**	Prostaglandin G/H synthase 2	−13.7	**3UA1**	Cytochrome P450 3A4	−11.4
**1NO6**	Protein Tyrosine Phosphatase 1B	−9.8	**3UA1**	Cytochrome P450 3A4	−13.7	**4EY7**	Acetylcholinesterase	−11.4
**3MDY**	Bone morphogenetic protein receptor type-1B	−9.8	**3O96**	RAC-alpha serine/threonine-protein kinase	−13.7	**5KDI**	Pleckstrin homology domain-containing family A	−11.4
**3EAH**	Nitric oxide synthase, endothelial	−9.7	**2PVR**	Casein kinase II subunit alpha	−13.7	**4D6Z**	Cytochrome P450 3A4	−11.3
**5E6H**	Lysine-specific demethylase 5A	−9.7	**3PAG**	Beta-lactamase	−13.7	**4IDT**	Mitogen-activated protein kinase kinase kinase 14	−11.3
**4R0A**	Toll-like receptor 8	−9.7	**4QBZ**	Toll-like receptor 8	−13.7	**3HOK**	Heme oxygenase 1	−11.3
**1NSI**	Nitric oxide synthase, inducible	−9.7	**3W3J**	Toll-like receptor 8	−13.6	**3NXQ**	Angiotensin-converting enzyme	−11.2
**1UHL**	Retinoic acid receptor RXR-beta	−9.7	**3QX3**	DNA topoisomerase 2-beta	−13.5	**2OC2**	Angiotensin-converting enzyme	−11.1
**2PQ5**	Dual specificity protein phosphatase 13 isoform B	−9.7	**4IDT**	Mitogen-activated protein kinase kinase kinase 14	−13.5	**4XII**	Cholinesterase	−11.1
**3O3U**	Advanced glycosylation end product-specific receptor	−9.7	**1JQE**	Histamine Methyltransferase	−13.5	**1ROS**	Macrophage metalloelastase	−11.1
**3UA1**	Cytochrome P450 3A4	−9.7	**4C8B**	Receptor-interacting serine/threonine-protein	−13.5	**2D0T**	Indoleamine 2,3-dioxygenase 1	−11.1
**1P0I**	Butyryl cholinesterase	−9.6	**4FM9**	DNA topoisomerase 2-alpha	−13.5	**4FYR**	Aminopeptidase N	−11.1
**2C66**	Amine oxidase [flavin-containing] B	−9.6	**4TUH**	Bcl-2-like protein 1	−13.5	**4R6A**	Toll-like receptor 8	−11.0
**3O96**	RAC-alpha serine/threonine-protein kinase	−9.6	**3EAH**	Nitric oxide synthase, endothelial	−13.4	**3W3J**	Toll-like receptor 8	−11.0

**PDB ID**: Protein Data Bank Identification Code. *. The runs were performed in triplicate.

**Table 2:** MMGBSA-based total binding free energies along with standard error of the mean (SEM).

Complexes					
Energy Components	Hsp70-1-ADP	Hsp70-1-DAST	CD40 Ligand-LKJ	CD40 Ligand-FB-71	CD40 Ligand-FB-28
Total Binding Free Energy (kcal/mol)	−25.96 ± 1.02	−10.23 ± 0.55	−17.56 ± 0.63	−10.53 ±0.50	−18.03 ± 0.67
Van der Waals Energy (kcal/mol)	−27.17 ± 0.94	−38.76 ± 0.46	−39.98 ± 0.58	−28.71 ± 0.72	−44.29 ± 1.30
Electrostatic Energy (kcal/mol)	−72.35 ± 2.54	−53.78 ± 1.09	−116.92 ± 1.98	111.31 ± 1.76	−34.88 ± 1.37
Polar solvation energy (kcal/mol)	77.92 ± 2.24	88.19 ± 0.83	144.13 ± 0.07	−89.34 ± 1.78	67.51 ± 1.34
SASA energy (kcal/mol)	−4.36 ± 0.092	−5.88 ± 0.035	−4.78 ± 0.50	−3.78 ± 0.13	−6.36 ± 0.14
ΔG gas	−99.5 ± 2.66	−92.55 ± 1.01	−156.90 ± 2.28	82.59 ± 1.82	−79.17 ± 1.63
ΔG solv.	73.56 ± 2.18	82.32 ± 0.83	139.34 ± 1.90	−93.13 ± 1.71	61.14 ± 1.25

**Table 3:** Changes in the relative mRNA expression for evaluated genes in transgenic *gfp*-reporter strains of *C. elegans* exposed to OBs.

OB Concentration (µM)		Relative Expression for GFP Transgenic Strains				
		Cellular Stress		Oxidative Stress		Metabolism
		*hsp-3*	*hsp-4*	*gpx-4*	*sod-4*	*gst-1*
DAST	0	1.00 ± 0.04	1.00 ± 0.06	1.00 ± 0.05	1.00 ± 0.04	1.00 ± 0.09
	50	1.04 ± 0,16	1.06 ± 0.13	1.19 ± 0.10	0.84 ± 0.26	0.86 ± 0.24
	100	1.00 ± 0.17	1.08 ± 0.19	1.60 ± 0.60 *	0.98 ± 0.38	1.62 ± 0.24 *
	250	1.21 ± 0.13	1.24 ± 0.19 *	2.09 ± 0.43 *	1.30 ± 0.29	1.04 ± 0.20
	500	1.55 ± 0.32 *	1.41 ± 0.20 *	3.20 ± 0.61 *	3.06 ± 0.90 *	2.11 ± 0.27 *
FB-71	0	1.00 ± 0.05	1.00 ± 0.00	1.00 ± 0.06	1.00 ± 0.11	1.00 ± 0.04
	50	1.11 ± 0.17	1.21 ± 0.19	1.03 ± 0.25	1.32 ± 0.36	0.97 ± 0.09
	100	1.14 ± 0.15	1.20 ± 0.14	1.17 ± 0.37	1.45 ± 0.48 *	0.96 ± 0.13
	250	1.14 ± 0.16	1.23 ± 0.31 *	1.35 ± 0.43	1.40 ± 0.23 *	1.03 ± 0.07
	500	1.46 ± 0.19 *	1.39 ± 0.18 *	1.70 ± 0.28 *	1.70 ± 0.29 *	1.08 ± 0.08
FB-28	0	1.00 ± 0.04	1.00 ± 0.02	1.00 ± 0.06	1.00 ± 0.05	1.00 ± 0.09
	50	1.05 ± 0.19	1.27 ± 0.23	1.04 ± 0.24	1.15 ± 0.24	1.23 ± 0.20
	100	1.06 ± 0.19	1.26 ± 0.34	0.93 ± 0.17	1.08 ± 0.15	1.27± 0.20
	250	1.10 ± 0.17	1.20 ± 0.28	0.98 ± 0.10	1.16 ± 0.13	1.28 ± 0.41
	500	1.12 ± 0.13	1.19 ± 0.23	0.96 ± 0.16	1.11 ± 0.14	1.20 ± 0.26

*. Significant difference compared to control (*p* < 0.05).

## Data Availability

Data are contained within the article and Supplementary Materials.

## Supplementary Materials

The following supporting information can be downloaded at: https://www.mdpi.com/article/10.3390/toxics12010051/s1, Table S1: Human proteins contained in PharmTargetDB with the 25 best "score" scores for DAST, FB-71 and FB-28; Table S2: Proteins taken from Environmental and Computational Chemistry Group (ECCG) data base.
